# Case report: cosegregation of a *TPM1* in-frame deletion (p.Lys7del) with familial non-compaction cardiomyopathy (NCCM)

**DOI:** 10.1007/s00392-023-02190-8

**Published:** 2023-04-17

**Authors:** Yvonne Hanel, Sven Dittmann, Klara Müller, Monica Elena Ioannou, Eric Schulze-Bahr

**Affiliations:** Department of Cardiovascular Medicine, ERN Reference Center GUARD-Heart, Institute for Genetics of Heart Diseases (IfGH), University Hospital Münster, Albert-Schweitzer-Campus 1 (D3), 48149 Münster, Germany

Sirs:

Left-ventricular non-compaction cardiomyopathy (NCCM) is a distinct cardiomyopathy with a compacted and non-compacted and hypertrabeculated myocardial layer of the LV wall with deep intertrabecular recesses [1, 2]. NCCM may occur either as an isolated phenotype, a non-syndromal entity (familial or in the setting with another inherited cardiomyopathies [dilated cardiomyopathy, DCM; hypertrophic cardiomyopathy, HCM; restrictive cardiomyopathy, RCM)] or as a part of a syndromal (mainly neuromuscular) disorder. Thereby, NCCM is etiologically diverse and resembles an incomplete myocardial compaction development during embryogenesis or reversible as an acquired entity, e.g. during pregnancy or in the athlete’s heart [2]. Accordingly, clinical signs and symptoms of NCCM may vary from incidental imaging findings up to severe arrhythmias, congestive heart failure, thromboembolic complications and sudden cardiac death [2].

Due to its potential occurrence with other inherited cardiomyopathies, common genes for NCCM have been meanwhile evaluated as disease-causing (e.g. truncating pathogenic variants in *MYH7, ACTN2* and *PRDM16* or exonic deletions in *RYR2*), or for NCCM in the setting with DCM (e.g. truncating pathogenic variants in *TTN or RBM20*), or with HCM (*MYBPC3*, *MYH7, TNNT2, ACTC1* or *TPM1*) [3]. Within these, the *TPM1* gene encodes for the sarcomeric protein α-tropomyosin, an α-helical coiled coil dimer [4] that binds to the actin filament and is significantly involved in the physiologic stability of actin to the contractile filaments in a calcium-dependent interaction of actin and myosin during cardiac muscle contraction [4]. So far, mutations in *TPM1* are mostly identified in patients with HCM (ClinGen: as definitive disease-causing gene) and DCM (ClinGen: with moderate disease-evidence) but are rarely found in patients with a familial, non-syndromal NCCM [4]. Recently, a rare variant burden excess for NCCM cases versus gnomAD controls has been noted [5]. Therefore, *TPM1* currently resembles a candidate gene with at least moderate causality evidence for NCCM ([5]; ClinGen: not curated so far). Of note, the gene is relatively tolerant for a loss-of-function (gnomAD: pLoF o/e ratio > 0.35) that resulted in a mostly uncertain or benign variant classification (e.g. ClinVar) for truncating or structural variants in any cardiomyopathy form.

Here, we report on a 3-generation family with autosomal dominant NCCM sharing an unpublished heterozygous *TPM1* in-frame deletion (p.Lys7del) being located in a conserved lysine repeat motif of the α-tropomyosin (Table 1).

**Table 1:** Clinical features of family members with left-ventricular non-compaction cardiomyopathy (NCCM) and a co-segregating, heterozygous in-frame deletion in the *TPM1* gene

Pedigree ID	Age at diagnosis	Cardiac symptoms	ECG features	Cardiac imaging
V-II	15 years	None	SR, 109 bpm, incomplete RBBB, P-mitrale	CHD: VSD, spontaneous closure within the first years of life; atrial septal defect of primum type, surgical correction at 5 years Cardiac MRI (at 14 years): normal LV and RV dimensions and functions, LV-EF 62% (normal: > 57%), LV-EDV 74 ml/m^2^ (< 104), LV mass 59 g/m^2^, no LV hypertrophy, normal atrial sizes, no LGE; hypertrabeculari-sation and NC/C of 3.0 in the apical septal and lateral LV wall, compatible with NCCM
V-III	9 years	None	Atrial rhythm (negative P-waves in leads II, III), 80 bpm, complete RBBB (110 ms)	Cardiac MRI (at 9 years): normal LV and RV dimensions and functions, LV-EF 55% (normal: > 55%), LV-EDV 99 ml/m^2^ (< 104), LV mass 57 g/m^2^, no LV hypertrophy, normal atrial sizes, no LGE; mild hypertrabecularisation and NC/C of 2.2 in the apical LV wall, suspicious for NCCM
IV-II	43 years	None	SR, 73 bpm, flat T-waves, in V4-V6 preterminal negative. Exercise ECG (6.4 METS, 193 bpm at maximum) unremark-able	Cardiac MRI (at 43 years): borderline LV, normal RV dimension and function, LV-EF 49% (normal: > 49%), LV-EDV 93 ml/m^2^ (< 93), LV mass 45 g/m^2^ (30–59), no LV hypertrophy, normal atrial sizes, no LGE; extended hypertrabe-cularisation and NC/C of 2.8 in the LV wall, compatible with NCCM
V-I	22 years	None	SR, 69 bpm, in V4-V6 biphasic T-waves. Exercise ECG (7.3 METS, 171 bpm at maximum) unremark-able	Cardiac MRI (at 22 years): borderline LV, normal RV dimension and function, LV-EF 51% (normal: > 55%), LV-EDV 96 ml/m^2^ (< 103), LV mass 56 g/m^2^ (30–59), no LV hypertrophy, normal atrial sizes, no LGE; hypertrabecularisation and NC/C of 2.9 in the apical LV wall, compatible with NCCM

CHD, congenital heart disease; SR, sinus rhythm; bpm, beat per minutes; RBBB, right bundle branch block; MRI, magnetic resonance imaging; LV, left ventricle; RV, right ventricle; LGE, late gadolinium enhancement; NC/C, ratio of non-compacted vs. compacted LV wall

**Fig. 1:**
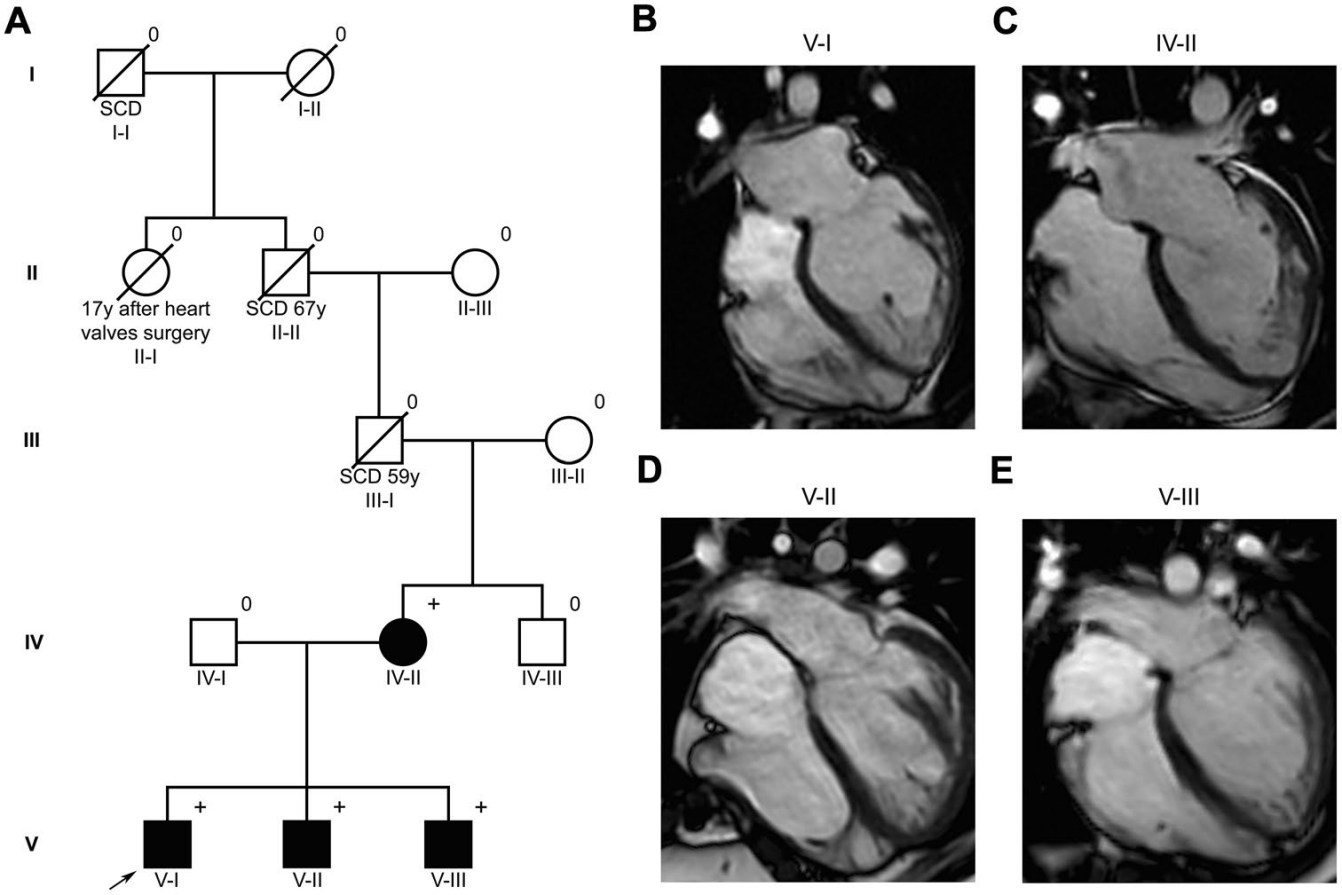
**A** Pedigree of the analysed family; the index patient is marked by an arrow; dark individuals: affected by NCCM; + : mutation carrier; 0: no DNA for analysis available; **B**–**E** Cardiac magnetic resonance images (four chamber view) of the index patient (pedigree number V-I) (**B**), his mother (pedigree number) IV-II (**C**) and his two brothers (pedigree numbers V-II and V-III) **D**–**E** showing all a non-compacted LV myocardium

The index patient was a 22 years-old man (Fig. 1 A, V-I) who was hospitalised after a car accident. He presented with T-wave inversion in V4 to V6 in the surface electrocardiogram. The patient did not show any angina pectoris complaints, arrhythmic events or syncope and was previously in a good, sportive state.

Transthoracic echocardiography showed a global mildly to moderately reduced left LV ejection fraction (LV-EF 45%) with global hypokinesis, a still normal diastolic function together with a pronounced myocardium with numerous trabeculations in the apical area of the central and apical segments of the left ventricle. Cardiac magnetic resonance imaging (Fig. 1B) verified the pronounced LV trabeculations (ratio non-compacted-to-compacted myocardium: 2.9) and confirmed the diagnosis of an isolated NCCM with a mildly reduced LV function. A baseline therapy with metoprolol was started.

With regard to the clinical presentation of the index patient and a positive maternal family history of sudden cardiac death (grandfather, great-grandfather and great-great-grandfather of the index patient, Fig. 1A), further cascade examination of the family was initiated. Hereby, the mother of the index patient (Fig. 1A, IV-II) was diagnosed to have a NCCM by cardiac magnetic resonance imaging (Fig. 1C), but with a normal systolic LV function and LV dimensions. Further, the two younger sons (15 years, Fig. 1A V-II; 9 years, Fig. 1A V-III) also had an apical hypertrabeculation of the LV (Fig. 1D–E) together with normal LV systolic function and without hypertrophy, altogether compatible with moderate NCCM phenotype, respectively. The diagnostic criteria for NCCM are still under discussion. Currently, the German non-compaction registry uses a combination of the so-called Zürich and Vienna criteria, which on the one hand focuses on the ratio of compacted and non-compacted layers and on the other hand on the number of trabeculation [6, 7].

Genetic evaluation by applying next-generation sequencing with a multi-gene panel set of > 70 cardiomyopathy genes (for DCM, HCM) revealed a predicted in-frame deletion of the lysine 7 residue (NM_001018005.1:c.20_22del; p.Lys7del) in the α-tropomyosin gene (*TPM1*) which was found to co-segregate with the familial disease. The variant classification was a likely pathogenic one (ACMG class 4 for NCCM; absent in controls/gnomAD (0%), PM1, PM2, PP1, PS4_supportive, PM4_supportive).

One son (V-II) had an atrial septal (ASD) as well as a ventricular septal defect (VSD) in addition to NCCM, which is in line with one study on TPM1 done in chicken. As TPM1 is important for the heart development and a knockdown of TPM1 has been reported in deficient atrial septation and ventricular trabeculae formation in chicken embryos, it appears likely that the identified *TPM1* variant indeed cause the inherited cardiac phenotype in the present family [8].

**Fig. 2:**
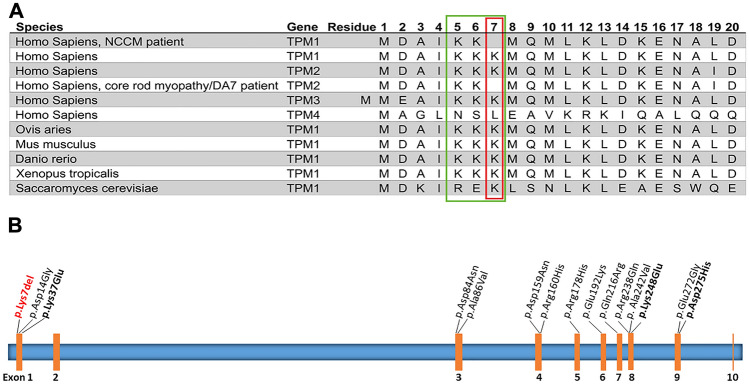
**A** Amino acid sequence homology of the TPM1 protein and other tropomyosins among different species: the conserved three lysine repeat motif is bordered in green and the deleted lysine 7 residue in red. **B** Distribution of reported non-synonymous 14 variants in *TPM1* and NCCM; in red, identified p.Lys7del; in unbold, reported sporadic or non-familial cases with NCCM; in bold, reported familial cases (Human Gene Mutation Database, January 2023)

So far, 81 pathogenic variants in *TPM1* are listed in HGMD, mostly associated with DCM or HCM. So far, 14 non-synonymous variants were associated with NCCM (listed in Human Gene Mutation Database (HGMD^®^ professional) and only three of those are familiar variants (Fig. 2B). In previous studies that analysed mainly isolated patients with LVNC/NCCM, only a small fraction showed variants in *TPM1* (< 2%) [9–12]. Due to the small number of publications so far, non-truncating variants in *TPM1* have been recently considered as a potential disease-causing gene for NCCM [5] and, moreover, a case excess vs. controls in gnomAD of rare *TPM1* variants has been shown [3]. Currently, *TPM1* has been thereby suggested as definitive gene for NCCM [5]. The deleted lysine residue is located in a repeat of three lysines which is highly conserved among different tropomyosin isoforms (TPM1, TPM2, TPM3) and among different species (Fig. 2). In TPM2, loss of the corresponding lysine 7 residue was found in a congenital myopathy [13] disrupting the N-terminus of β-tropomyosin and altering the β-tropomyosin dimer formation as well as the interactions with other molecules [13]. We speculate hereby that the mutant residue 7 in TPM1 may similarly also alter its protein function, e.g. by impairing its binding to leiomodin-2 (Lmod2) and tropomodulin-1 (Tmod1) which are both part of the thin sarcomeric filament and thereby critical for cardiac muscle function [14]. Variants of closely located residues of the N-terminus of α-tropomyosin (p.Met8Arg, p.Lys15Asn) have been found in DCM patients [15, 16]. These mutations disrupt formation of coiled coil domain at the N-terminal region of α-tropomyosin and, therefore, the interaction with Tmod1 and Lmod2 and possible other interaction partners [14]. Of note, the p.Met8Arg mutation in TPM1 is also associated to nemaline myopathy when it occurs in the corresponding residue of TPM3 [17].

In summary, there are only a few reports linking non-synonymous mutations in the *TPM1* gene to non-syndromal NCCM. Here, we report on another rare, but familial form of NCCM with a likely pathogenic *TPM1* in-frame deletion that may pave the way for further structure–function investigations among different tropomyosins. Since non-syndromal, familial NCCM cases are rare, this report may support the role of *TPM1* as a causative gene in NCCM.
